# Predicting Elevated TSH Levels in the Physical Examination Population With a Machine Learning Model

**DOI:** 10.3389/fendo.2022.839829

**Published:** 2022-02-24

**Authors:** Xinqi Cheng, Shicheng Li, Lizong Deng, Wei Luo, Dancheng Wang, Jin Cheng, Chaochao Ma, Luming Chen, Taijiao Jiang, Ling Qiu, Guojun Zhang

**Affiliations:** ^1^ Department of Clinical Diagnosis, Laboratory of Beijing Tiantan Hospital, Capital Medical University, Beijing, China; ^2^ Department of Laboratory Medicine, Peking Union Medical College Hospital, Chinese Academy of Medical Sciences, Beijing, China; ^3^ Institute of Systems Medicine, Chinese Academy of Medical Sciences & Peking Union Medical College, Beijing, China; ^4^ Suzhou Institute of Systems Medicine, Suzhou, China; ^5^ Guangzhou Laboratory, Guangzhou International Bio Island, Guangzhou, China

**Keywords:** elevated TSH level, machine learning model, antithyroid peroxidase-antibody (TPO-Ab), logistic analysis, free triiodothyronine (FT3) to thyroxine (FT4)

## Abstract

**Objective:**

The purpose of this study was to predict elevated TSH levels by developing an effective machine learning model based on large-scale physical examination results.

**Methods:**

Subjects who underwent general physical examinations from January 2015 to December 2019 were enrolled in this study. A total of 21 clinical parameters were analyzed, including six demographic parameters (sex, age, etc.) and 15 laboratory parameters (thyroid peroxidase antibody (TPO-Ab), thyroglobulin antibody (TG-Ab), etc.). The risk factors for elevated TSH levels in the univariate and multivariate Logistic analyses were used to construct machine learning models. Four machine learning models were trained to predict the outcome of elevated TSH levels one year/two years after patient enrollment, including decision tree (DT), linear regression (LR), eXtreme Gradient boosting (XGBoost), and support vector machine (SVM). Feature importance was calculated in the machine learning models to show which parameter plays a vital role in predicting elevated TSH levels.

**Results:**

A total of 12,735 individuals were enrolled in this study. Univariate and multivariate Logistic regression analyses showed that elevated TSH levels were significantly correlated with gender, FT3/FT4, total cholesterol (TC), TPO-Ab, Tg-Ab, creatinine (Cr), and triglycerides (TG). Among the four machine learning models, XGBoost performed best in the one-year task of predicting elevated TSH levels (AUC (0.87(+/- 0.03))). The most critical feature in this model was FT3/FT4, followed by TPO-Ab and other clinical parameters. In the two-year task of predicting TSH levels, none of the four models performed well.

**Conclusions:**

In this study, we trained an effective XGBoost model for predicting elevated TSH levels one year after patient enrollment. The measurement of FT3 and FT4 could provide an early warning of elevated TSH levels to prevent relative thyroid diseases.

## Introduction

Thyroid-stimulating hormone (TSH) is secreted by the pituitary gland and plays an important role in maintaining normal thyroid functions. An elevated TSH level is usually a signal of illness, such as hypothyroidism and Hashimoto thyroiditis (1, 2). Additionally, elevated TSH levels are risk factors for many pathological states. For instance, a recent study revealed that high TSH levels predicted long-term mortality in adults ever treated for hypothyroidism (3). Another study proved that a high TSH level was an independent risk factor for differentiated thyroid cancer and could contribute to the initiation of thyroid carcinogenesis (4). Since an elevated TSH level is a risk factor for thyroid diseases, accurate prediction of TSH levels can promote early, preventative intervention for high-risk patients and warn them to keep their eyes on potential thyroid diseases. Additionally, it can be seen from our dataset that when subjects first came to do the physical examination, their TSH levels were usually normal. Therefore, it is essential to predict elevated TSH levels over 1–2 years after patient enrollment.

As a data-driven technique, machine learning has been widely applied in constructing clinical prediction models in recent years. Machine learning models have also been used in predicting elevated TSH levels. Abou-Samra (5) chose seven clinical parameters (TPO antibodies, sex, etc.) as input to train machine learning models to predict whether TSH is normal, suppressed, or elevated. However, their prediction model was unable to predict future TSH levels. The purpose of this study was to construct a machine learning model to predict elevated TSH levels at a long time after physical examination based on patients’ demographic information and commonly measured laboratory parameters.

## Materials and Methods

### Study Population

Data were obtained from individuals who underwent physical examinations and laboratory tests at Peking Union Medical College Hospital from January 2015 to December 2019. Inclusion criteria: (1) individuals with physical examinations and laboratory tests; (2) individuals with normal TSH, FT3 (free triiodothyronine), and FT4 (free tetraiodothyronine) in the first year. The exclusion criteria were as follows: (1) patients without follow-up; (2) patients with a high incidence of missing data (more than 50%); and (3) patients with suppressed TSH levels. Subjects with a history of thyroid diseases or/and receiving related treatment are usually followed in the thyroid department instead of in the physical examination center, for which we did not exclude such subjects.

### Clinical and Laboratory Assessments

Weight and height were measured with the participants dressed in light clothes without shoes using a digital scale. Systolic blood pressure and diastolic blood were measured after a rest of 15 minutes, which was repeated three times on the right arm. In addition, the mean value of the three results was adopted. Blood samples for laboratory tests were extracted from patients after 8 hours of fasting. The tests included the following: alanine aminotransferase (ALT), total protein (TP), albumin (Alb), creatinine (Cr), urea, glucose (Glu), uric acid (UA), total cholesterol (TC), triglycerides (TG), high-density lipoprotein cholesterol (HDL-C) and low-density lipoprotein cholesterol (LDL-C).

The Tg-Ab and TPO-Ab measurements were performed using Roche kits and included reagents (Cobas e601, Roche Diagnostics, Mannheim, Germany) according to the manufacturer’s instructions. Serum levels of FT3, FT4, and TSH were measured with an ADVIA Centaur XP automatic chemiluminescence immunoassay analyzer (Siemens Healthineers, Erlangen, Germany) using the supplied reagents and calibrators.

The clinical variables involved in this study are the common items of physical examination in the hospital. In addition, these variables can affect or be affected by thyroid functions and thyroid hormone levels. For FT3, FT4, total triiodothyronine, and total thyroxine were all closely related to the hypothalamic-pituitary-thyroid axis, which would directly influence TSH physically. We did not use these clinical parameters as input of the Logistic analyses. It is noteworthy that the ratio of FT3 to FT4 was included in the logistic analysis and subsequent prediction models, which could indicate the influence of peripheral deiodination on future elevated TSH levels among euthyroid population.

### Statistical Analysis

Categorical variables are presented as percentages. Continuous variables are presented as medians (interquartile range). Wilcoxon rank-sum test was used for comparison of continuous variables. Univariate and multivariate Logistic regression analyses were performed to assess risk factors for elevated TSH. The odd ratios (ORs) and 95% confidence intervals (CIs) were calculated. The above statistical analysis was performed by R (version 3.6.0). P < 0.05 was considered statistically significant.

### Development of Machine Learning Models to Predict the Events of Elevated TSH

The Logistic regression analysis identified seven clinical variables as risk factors: gender, TPO-Ab, Tg-Ab, FT3/FT4, CR, TC, and TG. These seven clinical variables were used as the input of the machine learning models.

This prediction task was performed as follows: the input was the seven clinical variables from subjects when they first underwent the physical examination and laboratory tests, as well as the time of the event (TSH of a patient turned elevated); the output was a determination of whether a subject’s TSH level was elevated at a specific time (1 year or 2 years). In this prediction task, TSH (mIU/L) was classified into normal TSH (0.38–4.94) and elevated TSH (>4.94) (6), represented in the data frame as 0 and 1, respectively. The categorical variable gender in the data frame was converted into indicator variables. All the variables were normalized using the data normalization function in the preprocessing module of the Scikit Learn package.

In this study, we trained and evaluated four supervised machine learning models to predict TSH levels: decision tree (DT), linear regression (LR), eXtreme Gradient boosting (XGBoost), and support vector machine (SVM). In the modeling process, 5-fold cross-validation was adopted, in which patients were divided randomly into five subgroups with similar event rates. To develop the derivation cohort, four subgroups were combined. The other subgroup was taken as the validation set, and this procedure was repeated five times to ensure that every subgroup could serve as the validation set.

### Evaluation of the Machine Learning Models

Receiver operating characteristic (ROC) curves were utilized to evaluate model performance by calculating the area under the curve (AUC). Additionally, feature importance was estimated to explain how important a feature was in predicting elevated TSH levels.

The development and evaluation of machine learning models were conducted in Python version 3.8 (https://www.python.org) with Numpy, Scikit Learn, matplotlib, and XGBoost.

## Results

### Characteristics of the Patients Enrolled in This Study

A total of 12,735 patients were enrolled in this study. **Table 1** summarized the characteristics of the patients during follow-up with normal TSH and elevated TSH in the study population. There were significant differences in age, FT3 to FT4 ratio, TPO-Ab, Tg-Ab, Alb, Cr, UA, TC and TG between the two groups (all p < 0.05 for Wilcoxon rank-sum test).

**Table 1:** The characteristics of the patients during follow-up with normal TSH and elevated TSH in this study.

Characteristics	Normal TSH (n = 11,565)	Elevated TSH (n = 1,170)	Total (n = 12735)	P value (Wilcoxon rank-sum test)
Male (%)	5,308 (45.90%)	318 (27.18%)		
Age (years)	39 (31–49)	46 (38-54)	40 (32-50)	<0.001
FT3/FT4	2.53 (2.32-2.76)	2.63 (2.42-2.87)	2.55 (2.34-2.78)	<0.001
TPO-Ab (IU/ml)	13.91 (11.56-17.10)	15.08 (10.81-79.17)	13.96 (11.50-17.4)	0.002
Tg-Ab (IU/ml)	10.53 (10.0-13.93)	17.31 (11.68-192.17)	10.83 (10.2-14.83)	<0.001
ALT (U/L)	17.0 (12.0-25.0)	16.5 (13.0-23.0)	17.0 (12.0-24.0)	0.159
TP (g/L)	73.0 (71.0-76.0)	72.0 (69.0-74.0)	73.0 (70.0-76.0)	0.081
Alb (g/L)	47.0 (45.0-48.0)	43.0 (41.0-47.1)	46.0 (44.0-47.8)	<0.001
Cr (µmol/L)	70.3 (61.2-82.5)	66.1 (58.2-76.25)	69.0 (60.0-82.0)	<0.001
Urea (mmol/L)	4.47 (3.75-5.2)	4.41 (3.71-5.06)	4.46 (3.75-5.19)	0.123
Glu (mmol/L)	5.1 (4.8-5.5)	5.0 (4.7-5.3)	5.1 (4.8-5.4)	0.255
UA (µmol/L)	305.0 (255.1-371.0)	289.5 (242.5-341.0)	303.0 (254.5-369.0)	<0.001
TC (mg/dl)	4.57 (4.02-5.17)	4.78 (4.16-5.34)	4.59 (4.03-5.19)	<0.001
TG (mmol/L)	1.03 (0.73-1.56)	1.21 (0.88-1.68)	1.05 (0.74-1.57)	<0.001
HDL-C (mmol/L)	1.27 (1.06-1.51)	1.24 (1.05-1.47)	1.26 (1.06-1.50)	0.259
LDL-C (mmol/L)	2.83 (2.34-3.38)	2.88 (2.36-3.38)	2.83 (2.34-3.38)	0.439
Height (cm)	167 (161-174)	164 (159-171)	167 (161-173.2)	0.111
Weight (kg)	64 (56-75)	63 (57-72)	64 (56-74)	0.317
Diastolic blood pressure (mmHg)	117 (106-130)	119 (108-134)	117 (106-131)	0.910
Systolic blood pressure (mmHg)	72 (65-80)	73 (67-81)	72 (66-80)	0.453

Data was shown as median (interquartile range). FT3, free triiodothyronine; FT4, free thyroxine; TPO-Ab, antithyroid peroxidase autoantibody; Tg-Ab, anti-thyroglobulin antibody; ALT, alanine aminotransferase; TP, total protein; Alb, albumin; Cr, creatinine; Glu, glucose; UA, uric acid; TC, total cholesterol; TG, triglycerides; HDL-C, high-density lipoprotein cholesterol; LDL-C, low-density lipoprotein cholesterol.

### Risk Factors for Elevated TSH

As shown in **Table 2**, there was one significant protective factor for elevated TSH levels from the univariate Logistic regression analysis: TC (OR = 0.646, 95% CI: 0.603–0.706). There were six risk factors for elevated TSH levels, including gender, FT3/FT4, TPO-Ab, Tg-Ab, CR, TC, and TG. The most significant risk factor in the univariate Logistic regression analysis was FT3/FT4 (OR = 6.696, 95% CI: 4.668–9.605). From the multivariate Logistic regression analysis, there was one significant protective factor and six risk factors for elevated TSH levels, among which the most statistically significant risk factors were FT3/FT4 (OR = 3.170, 95% CI: 2.033-4.206) and Tg-Ab (OR = 2.746, 95% CI: 1.953-3.009), which indicated that the two factors were highly related with elevated TSH levels.

**Table 2:** Logistic regression analysis of the risk factors for elevated TSH.

Clinical Features	Univariate Logistic regression		Multivariate Logistic regression	
	OR (95% CI)	P value	OR (95% CI)	P value
Age	1.059 (0.943-1.188)	0.332	–	
Gender	1.932 (1.582-2.122)	0.001	1.517 (1.338-1.719)	0.006
FT3/FT4	6.696 (4.668-9.605)	<0.001	3.170 (2.033-4.206)	<0.001
TPO-Ab	3.088 (2.099-4.542)	<0.001	1.958 (1.659-2.378)	<0.001
Tg-Ab	2.001 (1.189-3.345)	0.029	2.746 (1.953-3.009)	<0.001
ALT	1.033 (0.875-1.214)	0.396	–	–
TP	1.157 (0.794-1.196)	0.405	–	–
Alb	0.950 (0.838-1.076)	0.278	–	–
CR	1.465 (1.347-1.600)	0.012	1.331 (1.178-1.467)	0.042
Urea	1.056 (0.935-1.192)	0.377	–	–
Glu	1.009 (0.902-1.129)	0.269	–	–
UA	0.974 (0.856-1.110)	0.368	–	–
TC	0.646 (0.603-0.706)	<0.001	0.637 (0.345-0.970)	0.027
TG	1.825 (1.623-2.503)	<0.001	1.749 (1.565-2.118)	<0.001
HDLC	1.151 (0.889-1.490)	0.585	–	–
LDLC	1.549 (0.895-2.180)	0.618	–	–
height	0.982 (0.971-1.185)	0.211	–	–
weight	1.001 (0.997-1.006)	0.168	–	–
Diastolic blood pressure	1.099 (0.969-1.187)	0.200	–	–
Systolic blood pressure	1.174 (0.997-1.195)	0.191	**-**	–

OR, odds ratio; CI, confidence interval; FT3, free triiodothyronine; FT4, free thyroxine; TPO-Ab, antithyroid peroxidase autoantibody; Tg-Ab, anti-thyroglobulin antibody; ALT, alanine aminotransferase; TP, total protein; Alb, albumin; Cr, creatinine; Glu, glucose; UA, uric acid; TC, total cholesterol; TG, triglycerides; HDL-C, high-density lipoprotein cholesterol; LDL-C, low-density lipoprotein cholesterol.

### Performance of the Machine Learning Models for Predicting the Events of Elevated TSH

**Figure 1:**
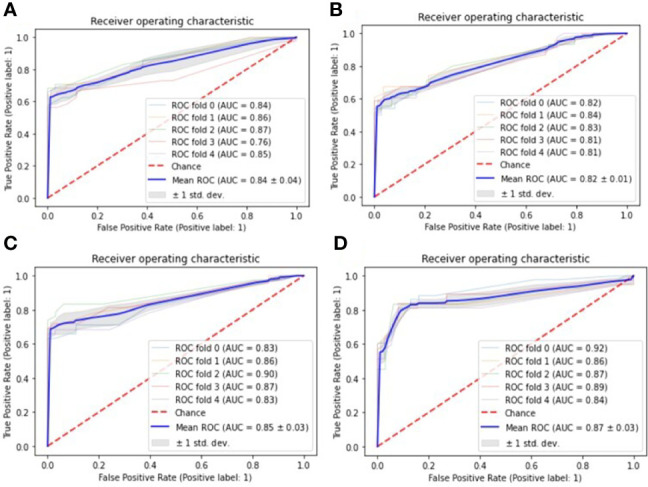
ROC curves for one-year prediction task, the Receiver Operating Characteristic (ROC) curves are used to evaluate the performance of four machine learning models with 5-fold cross-validation: **(A)** Decision tree; **(B)** Logistic regression; **(C)** Support vector machine; **(D)** eXtreme Gradient Boosting.

From **Table 3**, it can be seen that the machine learning model based on XGBoost achieved the best performance to predict the elevated TSH level in one year, which has the best accuracy [0.86 (+/- 0.07)] and the best AUC [0.87 (+/- 0.03)]. However, in predicting the elevated TSH level in two years, none of the models achieved good performance [the best AUC was 0.62 (+/- 0.05)]. **Figure 1** showed the ROC curves of the four machine learning models for predicting elevated TSH in one year.

**Table 3:** Performance of 5-fold cross-validation of the four machine learning models (mean ± SD).

One year	Accuracy	AUC	Two year	Accuracy	AUC
DT	0.80 (+/- 0.07)	0.84 (+/- 0.04)	DT	0.62 (+/- 0.07)	0.57 (+/- 0.05)
LR	0.79 (+/- 0.09)	0.82 (+/- 0.01)	LR	0.61 (+/- 0.03)	0.52 (+/- 0.04)
SVM	0.81 (+/- 0.08)	0.85 (+/- 0.03)	SVM	0.56 (+/- 0.04)	0.52 (+/- 0.07)
XGBoost	0.86 (+/- 0.07)	0.87 (+/- 0.03)	XGBoost	0.61 (+/- 0.06)	0.62 (+/- 0.05)

AUC, area under curve; DT, decision tree; LR, logisitic regression; SVM, support vector machine; Xgboost, eXtreme Gradient boosting.

### Feature Importance in the Prediction Model

**Figure 2:**
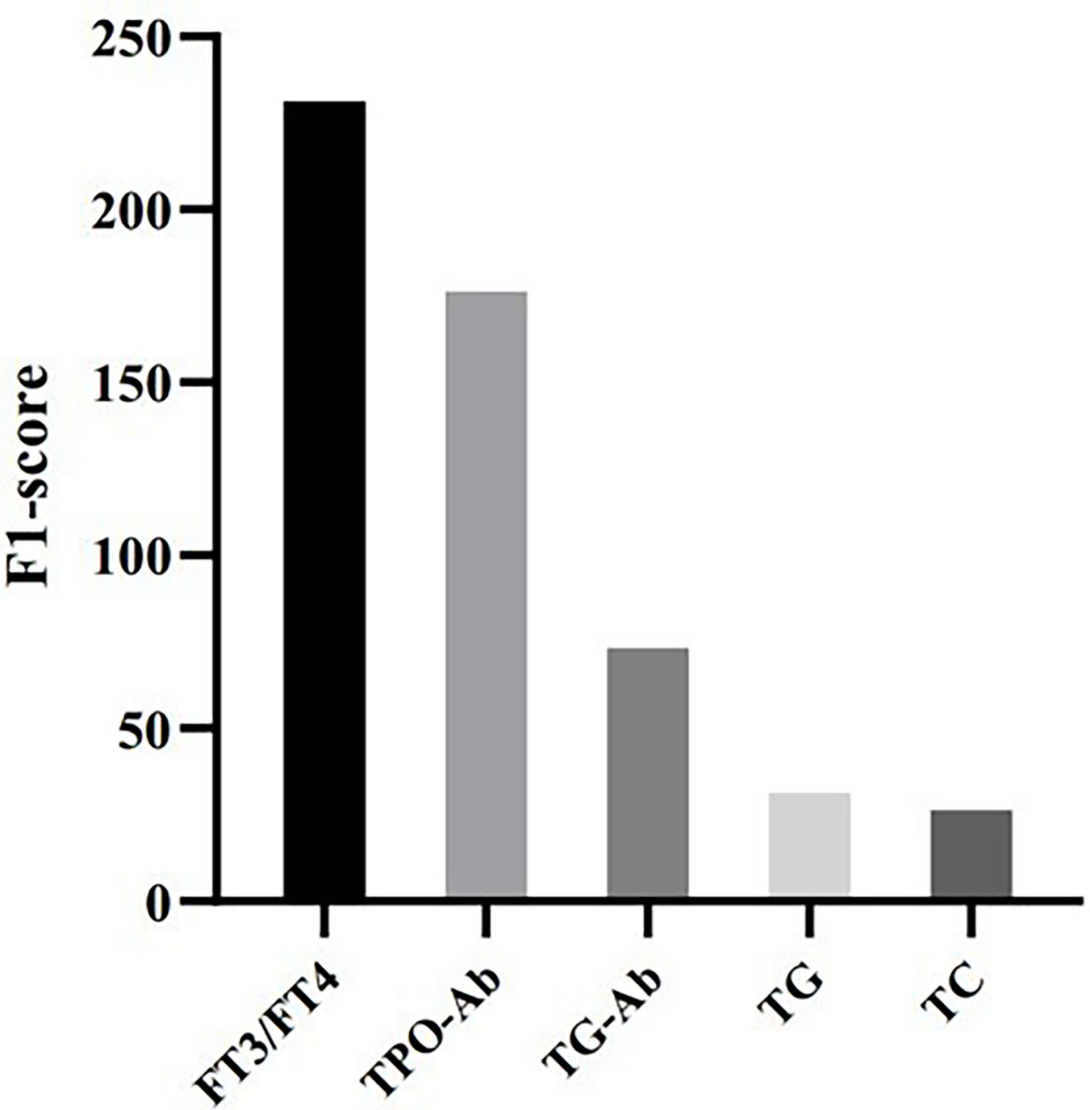
Feature importance in the XGBoost model.

To evaluate feature importance in the prediction model, we computed F1-scores for all the features used in the XGBoost model, which indicates how useful it is in the construction of the machine learning model. **Figure 2** shows that FT3/FT4 and TPO-Ab are the most important features in predicting elevated TSH, followed by Tg-Ab, TG, and TC.

## Conclusion

In summary, we developed an optimal XGBoost-based machine learning model for predicting elevated TSH levels over one year after patient enrollment.

## Discussion

In our study, a total of 12,735 subjects were enrolled with six demographic parameters and 15 commonly measured laboratory parameters. Univariate and multivariate Logistic regression analyses were conducted to assess risk factors for elevated TSH levels, which were used as the input of the machine learning prediction models. The output of the prediction models was if a TSH level was elevated one year/two years after patient enrollment. We trained four classic machine learning models to predict TSH levels: DT, LR, XGBoost, and SVM. The XGBoost-based model performed best (AUC (0.87 (+/- 0.03))) in predicting elevated TSH levels one year after the physical examination.

In multivariate Logistic analysis, elevated TSH levels were significantly correlated with gender, FT3/FT4, TPO-Ab, Tg-Ab, CR, TC, and TG. TC (7), gender (8), TPO-Ab (9), Tg-Ab (10), CR, and TG all have involvement in thyroid diseases. For example, males have a higher risk of recurrence in patients with papillary thyroid carcinoma than females (11). Another study evaluated the associations between thyroid antibodies (TPO-Ab and Tg-Ab) and the risk of preterm delivery (12). Recent research has shown some of the seven risk factors were independently related to TSH levels. For example, the oxidation of plasma total cholesterol (TC) could be raised by primary hypothyroidism, in which the TSH level was elevated, mainly because of an altered pattern of binding (13). The thyroid autoantibodies against thyroid peroxidase (TPO) and thyroglobulin (Tg) are the biochemical characteristics of Hashimoto’s thyroiditis, which is the most common cause of hypothyroidism (10). Also, hypothyroidism has been proved to be associated with increased serum creatinine (CR) (14). In particular, it is reported that the FT3/FT4 ratio would increase as TSH levels increase with the normal range of TSH levels (15). And this effect could be caused by increased deiodination of thyroxine (T4) to triiodothyronine (T3) in response to TSH. However, few studies have systematically demonstrated that the above clinical parameters are directly relevant to the TSH level.

Primary prevention is to take measures against pathogenic factors or risk factors when the disease has not occurred (16). It is an effective measure to eliminate the disease in the early stage. From the concept of primary prevention, we know that the core of primary prevention is to see the risk factors in advance. With the accurate prediction of potential elevated TSH, people could prevent the increase in TSH by early intervention (such as more iodine intake or proper exercise) to reduce the risk of other concurrent diseases. Additionally, owing to early warning, patients could be urged to perform check-ups more frequently to obtain timely and effective clinical intervention (17).

Among the four machine learning models in this study, XGBoost achieved the best accuracy and AUC. The XGBoost model is based on a gradient boosting decision tree characterized by high efficiency, flexibility, and portability and has achieved excellent performance in many clinical prediction problems (18). For example, Kai (19) used XGBoost to predict 30-day mortality for MIMIC-III patients with sepsis-3 with an AUC of 0.838. However, few studies have constructed machine learning models to predict TSH levels. In this study, compared with a one-year prediction task, XGBoost achieved poor performance in predicting TSH levels two years after enrollment. Generally, short-term prediction models are more stable than long-term models with the same input. The main reason is that the probability of affecting events may increase in a long time. In the well-performing XGBoost model, the ratio of FT3 to FT4 and TPOAb played the most important role in predicting TSH levels by calculating feature importance. Upon physical examination, some patients were TPOAb positive or/and TgAb positive, while TSH, FT3, and FT4 were normal, which would be neglected. Our prediction model will provide an available method to help predict TSH levels in the TPOAb/TgAb-positive cohort. In addition, measuring FT3 and FT4 to calculate the ratio of FT3 to FT4 is low-cost, which would greatly promote the preliminary screening of TSH elevation.

There were two limitations in this work. 1. An unbalanced dataset leads to a decline in the performance of machine learning models. There was a high proportion of negative samples (11565 in 12735). Additionally, for a few patients with suppressed TSH levels, we only analyzed the risk factors for elevated TSH levels and constructed the prediction model for high TSH levels. It is worth noting that the reduction in TSH is also a risk factor for diseases. 2. When excluding subjects, some conditions should be considered, such as thyroid-related basal diseases or pregnancy.

In the future, we aim to improve the performance of the prediction model by increasing the data scale; with extensive and balanced data, our prediction model could predict normal, elevated, and suppressed TSH levels of subjects over five years after the physical examination; multicenter clinical trials could further improve our algorithm performance.

## Data Availability Statement

The raw data supporting the conclusions of this article will be made available by the authors, without undue reservation.

## Ethics Statement

The studies involving human participants were reviewed and approved by Peking Union Medical College Hospital. Written informed consent for participation was not required for this study in accordance with the national legislation and the institutional requirements.

## Author Contributions

GZ designed the research. XC wrote the manuscript. TJ and LD designed the analytical strategies. SL performed data analyses. LC performed data preprocessing. WL, DW, JC, and CM performed data collection. LQ supervised this research. All authors contributed to the article and approved the submitted version.

## Conflict of Interest

The authors declare that the research was conducted in the absence of any commercial or financial relationships that could be construed as a potential conflict of interest.

## Publisher’s Note

All claims expressed in this article are solely those of the authors and do not necessarily represent those of their affiliated organizations, or those of the publisher, the editors and the reviewers. Any product that may be evaluated in this article, or claim that may be made by its manufacturer, is not guaranteed or endorsed by the publisher.
